# Insulin Resistance in Type 1 Diabetes: Concepts and Implications for Therapy

**DOI:** 10.1111/1753-0407.70126

**Published:** 2025-07-18

**Authors:** Zachary Bloomgarden, Domenico Accili

**Affiliations:** ^1^ Department of Medicine, Division of Endocrinology, Diabetes and Bone Disease Icahn School of Medicine at Mount Sinai New York New York USA; ^2^ Department of Medicine Columbia University Vagelos College of Physicians & Surgeons New York New York USA

Many people with type 1 diabetes (T1D) appear to develop insulin resistance. In a 6‐year post‐Diabetes Control and Complications Trial (DCCT) follow‐up of intensive treatment participants, comparison of the 61 having a family history of T2D with the 521 not reporting such a history showed BMI increasing by 3.8 versus 2.9 kg/m^2^, insulin requirement 0.73 versus 0.66 units/kg, and triglyceride 92 versus 76 [1].

Weight gain appears to be an important mediator of this insulin resistance. In the Pittsburgh Epidemiology of Diabetes Complications Study, adults with T1D had ~25% and 5% likelihood of having BMI 25–30 and ≥ 30, respectively, in 1986, with these figures increasing to ~35% and 25% by 2004 [2]; in the T1D Exchange Clinic Registry, by 2016–2018 more than two thirds of T1D adults age ≥ 26 were overweight or obese [3]. Comparing DCCT intensive treatment group participants in the top quartile of weight gain with those in the lower quartiles, insulin dose requirements, HbA1c, triglyceride, and BP were higher at DCCT close and 1‐ and 6‐years post‐trial, with those gaining the most weight being twice as likely to satisfy metabolic syndrome criteria at DCCT close, and 6 years later they were four times as likely to have metabolic syndrome [4]; in addition to the metabolic syndrome, metabolic‐associated fatty liver disease affects approximately one in five people with T1D [5].

Another potential cause of insulin resistance in T1D may be hyperglucagonemia. Along with insulin deficiency, T1D is associated with hyperglucagonemia, with loss of insulin‐induced glucagon suppression, and with increased α‐cell mass [6]. Patients with T1D appear insensitive to the glucagon‐suppressive effects of glucose and GLP‐1, the latter potentially an indirect effect of decreased endogenous insulin secretion or, perhaps, a secondary state of insulin resistance. In addition to its action in increasing hepatic gluconeogenesis and glycogenolysis, glucagon accelerates hepatic amino acid metabolism and ureagenesis, with a potential physiologic feedback circuit in which amino acids such as alanine increase glucagon secretion, with glucagon then reducing amino acid levels; disruption of the liver–α‐cell axis may cause hyperaminoacidemia and hyperglucagonemia, which may in turn contribute to hyperglycemia [7].

Insulin resistance becomes present early in the course of T1D [8]. The insulin resistance in T1D is not explained by body mass index, body fat percentage, visceral fat, plasma lipids, or physical activity, and is also not fully explained by the degree of hyperglycemia [9]. An additional cause of insulin resistance may be the hyperinsulinemia associated with peripheral insulin administration [10].

Insulin resistance in people with T1D appears, like that in type 2 diabetes, to be associated with the development of atherosclerotic complications. Using a hyperinsulinemic‐euglycemic clamp to assess insulin sensitivity in a group of people with T1D and in non‐diabetic controls, insulin resistance was associated with obesity, hypertriglyceridemia, and elevated coronary artery calcium scores [11]. Intensive treatment DCCT participants with metabolic syndrome characteristics had higher carotid artery intima‐media thickness than those not showing these features [4]. Among 1375 participants in the entire DCCT cohort, 49 had evidence of insulin resistance both at baseline and at 18.5‐year follow‐up with quadrupling in likelihood of developing cardiovascular disease compared to those who were insulin‐sensitive throughout the period of observation [12]. Insulin resistance was also associated with coronary disease risk in a 10‐year follow‐up of adults developing T1D in childhood [13, 14]. Using an estimate based on waist circumference among people with T1D, insulin resistance tracks with both cardiovascular disease and all‐cause mortality [15, 16]. Insulin resistance in T1D may also be associated with microvascular complications. In a 7‐year follow‐up of 764 patients with T1D not initially having diabetic retinopathy, the likelihood of developing retinopathy was increased among those with evidence of insulin resistance based on fasting triglyceride and waist‐hip ratio [17].

Based on these considerations, the use of medications developed for type 2 diabetes (T2D) may be appropriate for selected individuals with T1D. We discussed this topic nearly a decade ago [18] and can mention a few updates. Surprisingly few clinical trials have been carried out to ascertain whether there is a benefit of physical activity in improving insulin sensitivity among persons with T1D, although the published analyses do suggest that glycemia and dyslipidemia improve [19]; such approaches should always be considered appropriate. Metformin has potential benefit in improving insulin sensitivity among youth with T1D [20] and may be associated with an improvement in glycemia [21].

Both glucagon‐like peptide‐1 receptor agonists (GLP‐1RA) and Sodium‐Glucose Cotransporter‐2 inhibitors (SGLT2i) are increasingly being used in the United States for T1D treatment [22], and population studies show modest improvements in glycemia with these agents [23, 24]. A meta‐analysis of 11 studies of persons with T1D, 1965 receiving a GLP‐1RA and 844 controls, showed a modest effect, with HbA1c reduction of 0.21%, 4.04 kg weight loss, and 5.73 unit/day reduction in insulin dose [25], and a similar effect is seen in meta‐analyses of trials of SGLT2i in T1D, albeit with increased likelihood of the recognized complication of diabetic ketoacidosis [26, 27].

Thus, a large subset of persons with T1D have insulin resistance, often in the setting of weight gain, and we note the intriguing evidence of glycemic benefit of a number of T2D treatments for people with T1D. Recognizing that complications of T1D may differ to some extent in pathogenesis from those of T2D, the important unanswered question is whether, as in T2D, the GLP‐1RA and SGLT2i might be associated with improvement in renal and cardiovascular outcomes among people with T1D.

## Conflicts of Interest

The authors declare no conflicts of interest.
